# The Effects of Multi-Strain Probiotic SLAB51 Intake on Aerobic Performance and Oxidative Stress Responses in Elite Athletes: A Randomized Crossover Trial

**DOI:** 10.3390/nu18193299

**Published:** 2026-10-08

**Authors:** Sang-Yong Lee, Sehwa Kim, Jae-Hyeon Park, Hyo-Jun Yun, Jiwun Yoon

**Affiliations:** Center for Sports Analytics and AI, Korea National Sport University, Seoul 05541, Republic of Korea; leerres9@naver.com (S.-Y.L.); kke86624@gmail.com (S.K.); jhpark@knsu.ac.kr (J.-H.P.)

**Keywords:** randomized, double-blind, placebo-controlled, crossover study, probiotics, elite athletes

## Abstract

Background/Objectives: Exercise performance and recovery depend on interactions between energy metabolism and redox regulation. SLAB51, a multi-strain probiotic, may influence oxygen-related metabolism and antioxidant responses, but evidence in elite athletes remains limited. This study aimed to evaluate the effects of SLAB51 supplementation on aerobic exercise capacity and oxidative stress responses in elite athletes. Methods: This randomized, double-blind, placebo-controlled crossover trial evaluated the effects of SLAB51 supplementation on aerobic exercise performance and oxidative stress in elite athletes. Participants received SLAB51 and placebo for one week each, separated by a two-week washout period. Assessments were performed at baseline, after a single intake, and after one week of intake. Exercise performance was evaluated using a treadmill-based Bruce protocol, and physiological and oxidative stress markers were measured. Data were analyzed using two-way repeated-measures analysis of variance (ANOVA), supplemented by post hoc linear mixed-effects models (LMMs) accounting for the crossover design. Results: The original ANOVA showed significant treatment × time interactions for exercise duration, duration above a respiratory exchange ratio (RER) of 1.0, and pre-exercise superoxide dismutase (SOD) activity, but not for maximal oxygen uptake, lactate removal, or malondialdehyde concentration. The additional post hoc LMM analysis also identified a significant treatment difference in exercise duration after one week (mean difference = 33.32 s, 95% CI: 17.42–49.22, *p* < 0.001), with significant differences in duration above RER 1.0 and SOD activity also observed after one week. Conclusions: These findings support the potential benefits of one-week SLAB51 supplementation for exercise duration in elite athletes, consistent with the additional crossover-adjusted analysis. Favorable differences in duration above RER 1.0 and pre-exercise antioxidant enzyme activity warrant further investigation.

## 1. Introduction

Exercise performance and recovery are influenced by the interaction between energy metabolism and redox regulation. During exercise, oxygen utilization and aerobic energy production are essential for the sustained supply of adenosine triphosphate (ATP) and the maintenance of exercise performance [1,2,3,4]. However, increased oxygen consumption is also accompanied by the generation of reactive oxygen species (ROS), and excessive ROS production may contribute to oxidative stress, muscle fatigue, cellular damage, and delayed post-exercise recovery [5,6,7]. At the same time, ROS do not function solely as mediators of cellular damage. At physiological levels, they also act as important signaling molecules involved in mitochondrial biogenesis and the activation of antioxidant defense systems [8].

Therefore, maintaining an appropriate balance between ROS production and antioxidant defense is important for physiological adaptation to exercise, as both excessive ROS production and excessive suppression may impair adaptive responses [7,9]. In this context, the gut microbiota has received increasing attention as a potential modulator of metabolic and redox homeostasis [10,11]. Probiotic supplementation may influence these physiological processes by modulating the intestinal environment and host metabolic responses, and previous studies have suggested potential effects on metabolic regulation and oxidative stress modulation [12]. However, whether these effects translate into measurable changes in exercise performance, particularly in highly trained athletes, remains insufficiently understood.

SLAB51 is a multi-strain probiotic formulation that has been investigated for its potential effects on metabolic and antioxidant-related pathways. Previous studies using a bacterial lysate derived from SLAB51 have suggested that it may influence hypoxia-related responses in intestinal epithelial cells, including regulation of the hypoxia-inducible factor-1α (HIF-1α) pathway and alterations in cellular oxygen consumption and glycolytic metabolism [13]. In addition, SLAB51 has been reported to exert antioxidant-related effects through pathways involved in cellular protection against oxidative stress, including activation of the SIRT1 pathway in preclinical models [14]. These findings provide a potential biological rationale for investigating whether SLAB51 may influence physiological responses relevant to exercise.

Indeed, a number of preclinical and clinical studies have documented the physiological effects of SLAB51. Baldassarre et al. [15] reported significant increases in oxygen saturation (SaO_2_) and the ratio of SaO_2_ to the fraction of inspired oxygen (FiO_2_) following SLAB51 administration in oxygen-treated preterm infants, suggesting possible improvements in physiological responses related to oxygenation. Ceccarelli et al. [16] reported that the administration of SLAB51 to patients with COVID-19 was associated with more favorable blood oxygenation parameters. Collectively, these findings suggest that SLAB51 may influence physiological processes related to oxygenation and redox regulation.

However, previous studies have primarily been conducted in clinical or pathological populations, such as preterm infants or patients with infectious diseases, and direct evidence in healthy adults, particularly elite athletes exposed to repeated high-intensity exercise, remains limited [14,15,16]. Furthermore, ROS in exercise settings function not merely as mediators of cellular damage but also as signaling molecules involved in mitochondrial biogenesis and antioxidant adaptation [8]. Therefore, the physiological effects of SLAB51 observed in pathological populations cannot be assumed to occur in the same manner under exercise conditions. Direct investigation in highly trained athletes is therefore needed to determine whether SLAB51 influences exercise performance and redox-related physiological responses.

Accordingly, this study aimed to investigate the effects of short-term SLAB51 supplementation on aerobic exercise performance and antioxidant and oxidative stress-related responses in elite athletes. In addition, physiological responses following a single intake and after one week of supplementation were compared to examine whether the responses differed according to supplementation duration. By examining SLAB51 in a highly trained athletic population, this study sought to extend the existing evidence, which has predominantly been derived from clinical and pathological populations, to the context of sports performance.

## 2. Materials and Methods

### 2.1. Study Design

**Figure 1:**
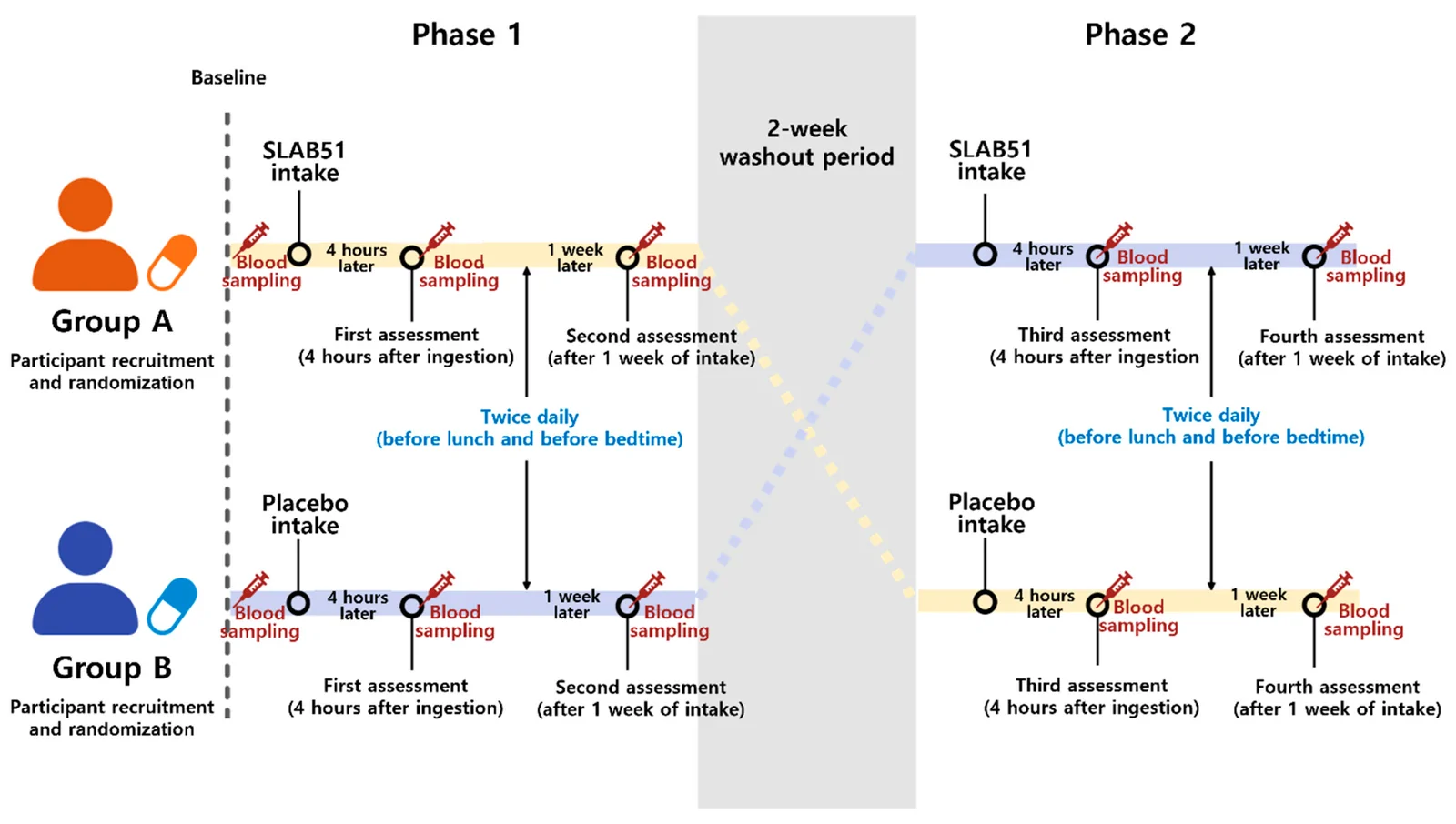
Randomized, double-blind, placebo-controlled, crossover trial design and measurement protocol.

This study was a randomized, double-blind, placebo-controlled, crossover clinical trial designed to evaluate the effects of SLAB51 supplementation on aerobic exercise capacity and oxidative stress responses in elite athletes. The trial was conducted at the Center for Sports Analytics and AI, Korea National Sport University, Seoul, Republic of Korea. Following the initial baseline assessment, participants were assigned to treatment-sequence Group A or Group B in a 1:1 ratio using a covariate-adaptive randomization method that considered sport type, body mass index (BMI), maximal oxygen uptake (VO_2_max), and blood lactate concentration as covariates [17]. The allocation sequence was generated and retained by an independent researcher using a computer-based covariate-adaptive randomization procedure implemented in Microsoft Excel 2024 (Microsoft Corporation, Redmond, WA, USA). Investigators responsible for participant enrollment did not have access to the allocation sequence, which remained concealed until each participant had been enrolled and assigned to a treatment sequence. The study consisted of two one-week intervention periods separated by a two-week washout period. The washout period was implemented to minimize potential residual effects from the preceding intervention. A two-week washout period has also been employed in a previous randomized, double-blind, placebo-controlled crossover trial investigating probiotic supplementation [18]. Accordingly, the two-week interval was considered a reasonable period for minimizing potential carryover effects. However, residual carryover effects cannot be completely excluded. During the first intervention period, Group A received SLAB51, whereas Group B received the placebo. Following the washout period, the interventions were crossed over: Group A received the placebo, whereas Group B received SLAB51. Participants and investigators responsible for intervention administration and outcome assessment were blinded to the treatment allocation. All assessments were conducted under identical environmental conditions. A single initial baseline assessment was obtained before randomization and any supplementation. This initial assessment was selected as the common untreated reference for both treatment conditions because later assessments could be influenced by familiarization or learning from repeated testing. During each intervention period, outcome measurements were performed 4 h after a single intake and after one week of supplementation. No separate pre-intervention baseline assessment was performed before the second intervention period. All post-supplementation measurements were conducted 4 h after supplement intake to ensure consistency in the timing of evaluation. The study protocol, including the planned outcome assessments and statistical analysis methods, was finalized prior to participant enrollment and data collection and was approved by the Institutional Review Board before the trial commenced. The overall study design and measurement protocol are presented in Figure 1. No important changes were made to the trial design or intervention procedures after the trial commenced.

### 2.2. Participants

**Figure 2:**
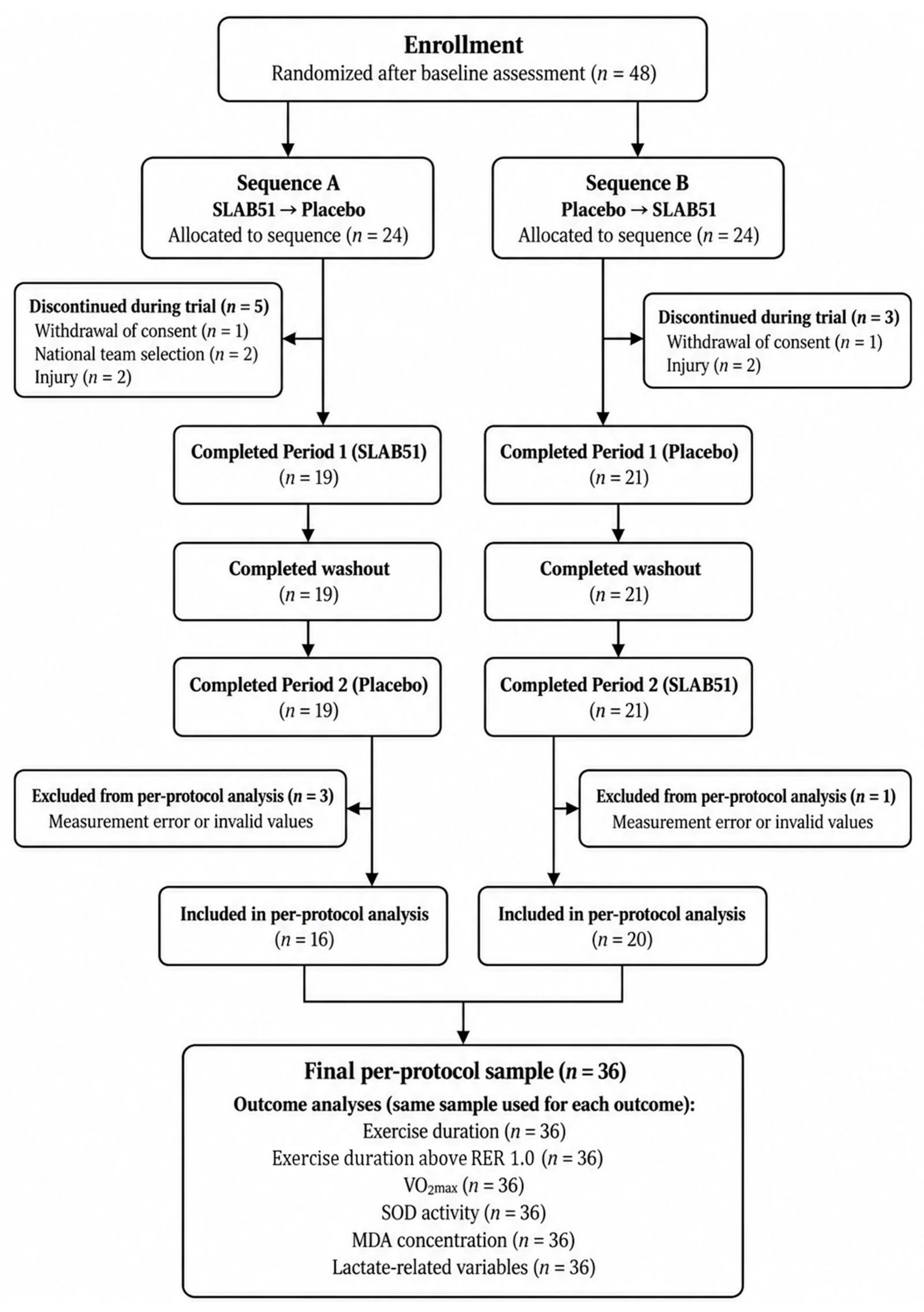
CONSORT crossover flow diagram of participant enrollment, allocation, follow-up, and analysis.

The participants in this study consisted of 48 elite athletes aged 18 years or older who were recruited using purposive sampling. The required sample size was estimated a priori using G*Power 3 software (version 3.1). In the absence of directly comparable previous studies investigating SLAB51 supplementation in elite athletes, a large effect size (Cohen’s *f* = 0.40) was assumed according to Cohen’s conventional effect-size criteria. With a significance level (α) of 0.05 and statistical power (1 − β) of 0.80, the minimum required sample size was estimated to be 40 participants. The target sample size was increased by 20%, from 40 to 48 participants, to allow for anticipated dropouts. The trial assessment period, including assessments of efficacy outcomes and adverse events, extended from 28 October to 19 December 2025. Before participation, all participants were provided with a comprehensive explanation of the purpose and procedures of the study, and written informed consent was obtained from each participant. The study protocol and informed consent forms were reviewed and approved by the Institutional Review Board of Korea National Sport University (IRB No. 1263-202509-BR-012-02; date of approval: 27 October 2025). The inclusion criteria were athletes who maintained regular training during the study period and had no cardiovascular, respiratory, or metabolic diseases. The exclusion criteria included refusal to provide informed consent, inability to perform normal exercise during the study, or the onset of health problems. A total of 48 participants were randomly assigned to treatment-sequence Group A (*n* = 24) or Group B (*n* = 24). During the trial, five participants in Group A discontinued because of withdrawal of consent (*n* = 1), national team selection (*n* = 2), or injury (*n* = 2), whereas three participants in Group B discontinued because of withdrawal of consent (*n* = 1) or injury (*n* = 2). Among the 40 participants who completed the trial, three participants from Group A and one participant from Group B were additionally excluded from the per-protocol analysis because one or more outcome measurements were considered unreliable due to technical or analytical problems. These included invalid blood-derived measurements associated with laboratory analysis problems and physiologically implausible VO_2_max values considered to be attributable to equipment- or measurement-related errors. Consequently, the final per-protocol analysis included 36 participants (Group A, *n* = 16; Group B, *n* = 20), which was below the initially estimated minimum sample size of 40. The higher-than-anticipated attrition and exclusion rate was therefore acknowledged as a limitation of the study. The participant enrollment, allocation, follow-up, and analysis process is presented in Figure 2.

### 2.3. Supplementation Protocol

**Figure 3:**
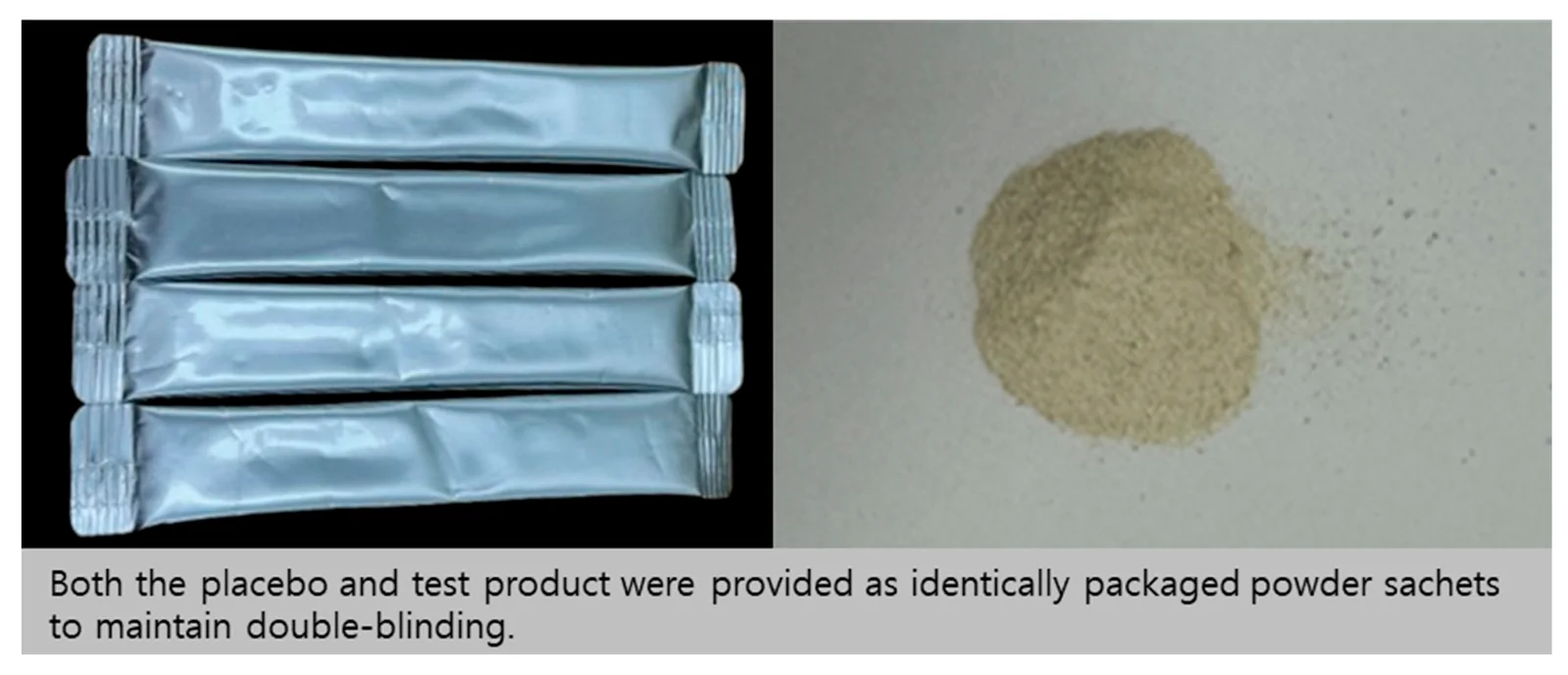
Appearance and packaging of the investigational product (SLAB51) and the placebo. Both the test product and the placebo were provided as identical packaged powder sachets in agreement with the double-blinded design of the study.

The investigational product utilized in this study was designated SLAB51, a multi-strain probiotic formulation (SLAB51, provided by Hecto Healthcare Co., Ltd., Seoul, Republic of Korea) composed of the bacterial strains *Streptococcus thermophilus* CNCM I-5570, *Bifidobacterium animalis* subsp. *lactis* CNCM I-5571, *Bifidobacterium animalis* subsp. *lactis* CNCM I-5572, *Lactobacillus acidophilus* CNCM I-5567, *Lactobacillus helveticus* CNCM I-5573, *Lactobacillus paracasei* CNCM I-5568, *Lactobacillus plantarum* CNCM I-5569, and *Lactobacillus brevis* CNCM I-5566. The investigational product was provided in individually packaged sachets, and the participants were instructed to take SLAB51 or a placebo twice daily on an empty stomach with water at each intake stage. The single serving size of the investigational product was set at four sachets, and the participants took it once before lunch and once before bedtime. Accordingly, the total daily intake was eight sachets, equivalent to a total of 1.6 × 10^12^ colony-forming units (CFU) per day. To monitor adherence, the daytime dose was administered at the laboratory under research staff supervision. The evening dose was administered at a designated location in the dormitory, where intake was confirmed and recorded by supervising staff. The placebo was meticulously crafted to mirror the investigational product in terms of appearance, color, and packaging. However, it was devoid of any probiotic ingredients. In order to maintain the integrity of the double-blind method, both the study participants and the researchers were unaware of the assignment of the investigational product or the placebo. Instead, the distribution and management of the investigational product were overseen by a separate researcher who was not directly involved in the study. The visual appearance and packaging of the investigational product and the placebo are depicted in Figure 3.

### 2.4. Experimental Procedures

**Figure 4:**
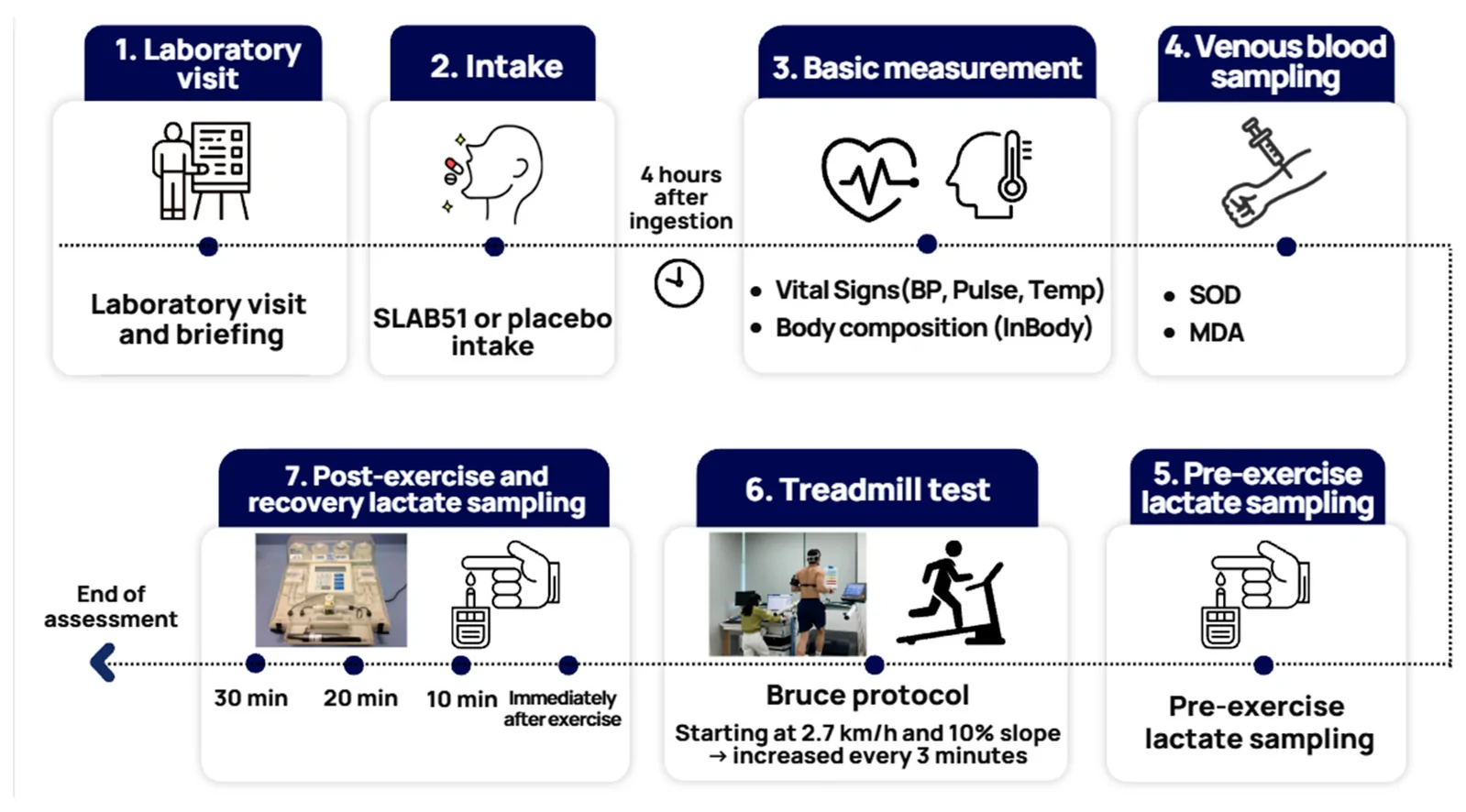
Protocol for exercise stress testing and measurement of physiological indicators.

All experiments were conducted under identical environmental conditions. Participants were instructed to abstain from vigorous exercise for at least 12 h before each assessment and to arrive at the laboratory in a fasted state. Upon arrival at the laboratory, participants received a brief explanation of the study purpose and procedures and then consumed either SLAB51 or a placebo according to their randomized group assignment. After the single intake, basic anthropometric and physiological measurements were conducted, including blood pressure, heart rate, body temperature, and body composition measurements (InBody 770, InBody Co., Ltd., Seoul, Republic of Korea). Subsequently, venous blood samples were collected for the analysis of antioxidant enzymes and oxidative stress markers. Venous blood samples were collected into 5.0-mL serum-separating tubes (SST). After collection, the tubes were gently inverted 8–10 times and allowed to clot in an upright position for at least 30 min. Samples were centrifuged within 1 h of collection at 3000 rpm for 10 min at 4 °C. The separated serum was aliquoted into 2.0-mL microtubes, with 1.0 mL allocated for each SOD and MDA analysis, and stored frozen at ≤−80 ± 10 °C until analysis. SOD activity was measured using a colorimetric method, whereas MDA concentration was measured using an ELISA-based method. Finger-stick blood samples were also collected to measure pre-exercise lactate concentrations. To evaluate exercise performance and oxygen metabolic responses, a graded exercise test was performed using the treadmill-based Bruce protocol on a COSMED T170 DE treadmill (COSMED srl, Rome, Italy). Before each test, the metabolic measurement system was warmed up and calibrated according to the laboratory calibration procedure, including flow-volume and gas calibration. Respiratory gas exchange was measured continuously using a breath-by-breath cardiopulmonary exercise testing system (Quark CPET, COSMED srl, Rome, Italy). Participants remained seated before testing, and the test was initiated after a stable resting condition was confirmed (heart rate < 100 beats/min and respiratory quotient of approximately 0.70–0.85). The Bruce protocol was initiated at a speed of 2.7 km/h with a 10% incline, with speed and incline increased every 3 min according to the standard protocol. The test was continued until volitional exhaustion and was terminated when the participant verbally indicated that he could no longer continue exercising. VO_2_, RER, heart rate, and exercise duration were continuously monitored throughout the test. To assess lactate responses to exercise, blood lactate concentrations were measured using a YSI 1500 SPORT Lactate Analyzer (YSI Inc., Yellow Springs, OH, USA) at five time points: before exercise, immediately after exercise, and at 10, 20, and 30 min during the recovery period. Adverse events were assessed non-systematically throughout the trial through participant self-report and investigator observation. The detailed experimental protocol is presented in Figure 4.

### 2.5. Outcome Measures

The outcome measures were classified into exercise performance indicators, antioxidant and oxidative stress indicators, and blood lactate responses (Table 1). Exercise performance indicators included VO_2_max, exercise duration, and exercise duration above RER 1.0. Exercise duration during the graded treadmill test was designated as the primary efficacy outcome because it directly reflected exercise performance under the standardized Bruce protocol. VO_2_max and exercise duration above RER 1.0 were classified as secondary exercise performance outcomes. Exercise duration was defined as the total time from the start of the Bruce protocol until voluntary exhaustion. Exercise duration above RER 1.0 was defined as the elapsed time from the first point at which RER exceeded 1.0 and remained above 1.0 for at least 10 consecutive seconds until exercise termination. Transient excursions above 1.0 that were not sustained for at least 10 s were not used to define the onset. Antioxidant and oxidative stress-related indicators included superoxide dismutase (SOD) activity and malondialdehyde (MDA) concentration, which were analyzed using venous blood samples collected before the treadmill exercise test. SOD activity, MDA concentration, and measured blood lactate concentrations were considered secondary outcomes. The derived lactate increase and lactate removal variables were considered exploratory post hoc outcomes. Blood lactate concentrations were measured before exercise, immediately after exercise, and at 10, 20, and 30 min during recovery. For calculation of the derived lactate variables, *L*_peak_ was defined as the highest observed lactate concentration among the post-exercise measurements. These measurements were obtained immediately after exercise and at 10, 20, and 30 min of recovery. *L*_peak_ was therefore not limited to the immediately post-exercise value alone [19]. Lactate increase and lactate removal were calculated to evaluate exercise-induced lactate accumulation and post-exercise recovery, respectively.

<Lactate Increase (mmol/L)>

*Lactate Increase* = *L_peak_* − *L_rest_* (1)

<Lactate Removal (%)>

(2) $$\mathrm{Lactate}\;\mathrm{Removal}\;(\%)=\left(1-\frac{L_{30\min}-L_{\mathrm{rest}}}{L_{\mathrm{peak}}-L_{\mathrm{rest}}}\right)\times 100$$

### 2.6. Statistical Analysis

A two-way repeated-measures analysis of variance (ANOVA) was conducted to examine the effects of treatment condition and measurement time on the study outcomes. Because all participants received both SLAB51 and placebo in the crossover trial, treatment condition (SLAB51 and placebo) and measurement time (baseline, after a single intake, and after one week of intake) were treated as within-subject factors. The treatment condition × measurement time interaction effect was also examined. To provide a common pre-randomization reference in view of potential changes associated with repeated testing, baseline was measured once for each participant, and the same baseline value was used under both treatment conditions in the original repeated-measures ANOVA. Although treatment and time were specified as within-participant factors, no additional constraint was specified to explicitly account for the perfect dependence arising from the use of the same baseline observation under both conditions. When a statistically significant main effect or interaction effect was identified, pairwise comparisons with Bonferroni adjustment were performed to determine differences between treatment conditions and measurement time points. Bonferroni adjustment was applied only to post hoc pairwise comparisons within each outcome and was not applied jointly across all study outcomes. The primary outcome omnibus test was not subject to multiplicity correction. The statistical significance level was set at *p* < 0.05. All statistical analyses were conducted using a participant-level per-protocol analysis set comprising 36 participants who completed both intervention periods. Among the 40 participants who completed the trial, four participants with one or more outcome measurements considered unreliable because of technical or analytical problems were excluded from the per-protocol analysis set. Consequently, otherwise valid outcome measurements from these participants were not retained in outcome-specific analyses. No imputation was performed for missing or excluded values. Participants were analyzed according to the treatment condition received during each intervention period. Blood lactate concentrations measured before exercise, immediately after exercise, and at 10, 20, and 30 min during recovery were treated as prespecified outcome measures. Lactate increase and lactate removal were calculated post hoc as additional derived outcomes using the measured lactate concentrations. Lactate removal (%) was calculated using the 30-min recovery lactate concentration according to Equation (2): [1 − (*L*_30 min_ − *L*_rest_)/(*L*_peak_ − *L*_rest_)] × 100. The lactate removal values therefore represent the percentage of exercise-induced lactate elevation removed by 30 min of recovery. Period and randomized sequence were not separately modeled in the original ANOVA. No subgroup analyses were conducted. All statistical analyses were performed using the Statistical Package for the Social Sciences (SPSS) for Windows, version 25.0. Data are presented as means and standard deviations (mean ± SD). An additional post hoc linear mixed-effects model (LMM) analysis was performed for exercise duration (primary outcome), exercise duration above RER 1.0, and pre-exercise SOD activity (secondary outcomes), using 144 post-randomization observations (excluding baseline) from 36 participants. Treatment, period, randomized sequence, assessment time (4 h after a single intake vs. one week), treatment × time, and period × time were included as fixed effects, with a participant-specific random intercept to account for within-subject dependence.

The treatment × time interaction allowed treatment contrasts to differ between the single-intake and one-week assessments, whereas period × time allowed assessment-time patterns to differ between intervention periods. The random-intercept model assumed a common covariance among observations from the same participant and conditionally independent residual errors with constant variance.

Models were fitted using restricted maximum likelihood (REML) with Satterthwaite degrees of freedom and Type III tests. Treatment contrasts (SLAB51 − placebo) at each assessment time were reported with 95% confidence intervals. Bonferroni adjustment was specified within each time point, with only one treatment comparison per time point; no joint adjustment across time points or outcomes was applied.

## 3. Results

### 3.1. Participant Characteristics

A total of 36 elite athletes completed all experimental procedures and were included in the final analysis. All participants were male competitive athletes. The study population consisted of 19 taekwondo athletes (52.8%), 12 field hockey athletes (33.3%), and 5 badminton athletes (13.9%). In this study, elite athletes were operationally defined as registered competitive athletes who regularly participated in structured sport-specific training and official competitions. The baseline physiological characteristics of the participants are summarized in Table 2.

### 3.2. Changes in Exercise Performance Indicators

#### 3.2.1. Changes in Exercise Duration According to SLAB51 Intake and Measurement Time Point

Exercise duration differed according to treatment condition and measurement time point, with a significant interaction effect between treatment condition and measurement time point (*p* = 0.002; Table 3). Post hoc analysis showed that, in the SLAB51 group, exercise duration significantly increased after single intake (*p* = 0.001) and after 1-week intake (*p* = 0.001) compared with baseline, with a further significant increase observed after 1-week intake compared with after single intake (*p* = 0.004). In the placebo group, exercise duration also significantly increased after single intake (*p* = 0.001) and after 1-week intake (*p* = 0.001) compared with baseline. However, no significant difference was observed between the single intake and 1-week intake time points (*p* = 0.088; Table 4).

#### 3.2.2. Changes in Exercise Duration Above RER 1.0 According to SLAB51 Intake and Measurement Time Point

Exercise duration above RER 1.0 differed according to treatment condition and measurement time point, with a significant interaction effect between treatment condition and measurement time point (*p* = 0.004; Table 5). Post hoc analysis showed that, in the SLAB51 group, exercise duration above RER 1.0 significantly increased after 1-week intake compared with baseline (*p* = 0.001) and after single intake (*p* = 0.004). However, no significant difference was observed between baseline and after single intake (*p* = 0.192). In the placebo group, no significant changes were observed across any measurement time points (Table 6).

#### 3.2.3. Changes in VO_2_max According to SLAB51 Intake and Measurement Time Point

VO_2_max did not differ significantly according to treatment condition or measurement time point, and no significant interaction effect between treatment condition and measurement time point was observed (*p* = 0.159; Table 7). Mean comparisons showed that VO_2_max in the SLAB51 group changed from 55.08 ± 5.49 at baseline to 56.50 ± 6.03 after single intake and 53.80 ± 6.34 after 1-week intake. Similarly, no significant changes across measurement time points were observed in the placebo group.

### 3.3. Changes in Antioxidant and Oxidative Stress Indicators (SOD and MDA)

#### 3.3.1. Changes in SOD According to SLAB51 Intake and Measurement Time Point

SOD differed according to treatment condition and measurement time point, with a significant interaction effect between treatment condition and measurement time point (*p* = 0.029; Table 8). Post hoc analysis showed that, in the SLAB51 group, SOD significantly increased after 1-week intake compared with baseline (*p* = 0.003) and after single intake (*p* = 0.001). However, no significant difference was observed between baseline and after single intake (*p* = 0.167). In contrast, no significant changes across measurement time points were observed in the placebo group (Table 9).

#### 3.3.2. Changes in MDA According to SLAB51 Intake and Measurement Time Point

MDA showed a significant main effect of measurement time (*p* < 0.001), but no significant main effect of treatment condition (*p* = 0.947) or treatment condition × measurement time interaction (*p* = 0.290; Table 10). Mean comparisons showed that MDA in the SLAB51 group temporarily increased from 36.54 ± 6.31 at baseline to 53.23 ± 25.37 after single intake, followed by a decrease to 49.95 ± 16.71 after 1-week intake. In contrast, the placebo group showed a gradual increase over time, ranging from 36.54 ± 6.31 at baseline to 47.82 ± 21.17 after single intake and 55.76 ± 21.17 after 1-week intake.

### 3.4. Changes in Lactate Removal (Lactate Reduction)

The reported lactate removal variable showed a significant main effect of measurement time (*p* < 0.001), but no significant main effect of treatment condition (*p* = 0.052) or treatment condition × measurement time interaction (*p* = 0.318; Table 11). Mean comparisons showed that lactate removal in the SLAB51 group gradually decreased from 86.37 ± 18.55 at baseline to 71.89 ± 10.56 after single intake and 68.67 ± 11.81 after 1 week. Similarly, the placebo group also showed a gradual decrease over time, ranging from 86.37 ± 18.55 at baseline to 74.78 ± 12.45 after single intake and 72.70 ± 10.78 after 1-week, with no substantial differences in the pattern of change between groups.

### 3.5. Post Hoc Crossover Analysis

The post hoc crossover analyses identified treatment × time interactions for total exercise duration (*F*(1, 103) = 7.084, *p* = 0.009), exercise duration above RER 1.0 (*F*(1, 103) = 4.105, *p* = 0.045), and pre-exercise SOD activity (*F*(1, 103) = 5.608, *p* = 0.020). After one week, SLAB51 was associated with greater total exercise duration by 33.32 s (95% CI: 17.42 to 49.22; *p* < 0.001), greater exercise duration above RER 1.0 by 56.99 s (95% CI: 18.71 to 95.26; *p* = 0.004), and higher SOD activity by 0.199 U/mL (95% CI: 0.037 to 0.361; *p* = 0.017) than placebo. No statistically significant treatment contrasts were observed after a single intake. SOD also showed period (*p* < 0.001) and period × time (*p* = 0.003) effects. The treatment contrasts are presented in Table 12.

## 4. Discussion

The purpose of this study was to determine the effects of short-term SLAB51 supplementation on aerobic exercise performance and antioxidant and oxidative stress-related responses in elite athletes. The principal findings were significant treatment condition × measurement time interactions for exercise duration, exercise duration above RER 1.0, and pre-exercise SOD activity. In contrast, no significant treatment-related interaction effects were observed for VO_2_max, MDA concentration, or lactate removal. Importantly, these findings should be interpreted by distinguishing changes from the common untreated baseline, responses potentially attributable to repeated-test familiarization, responses observed under the placebo condition, and differential responses between SLAB51 and placebo.

First, exercise duration increased from the common untreated baseline under both the SLAB51 and placebo conditions. Therefore, the improvement observed within the SLAB51 condition alone should not be interpreted as sufficient evidence of a treatment-specific effect. The significant improvement also observed under the placebo condition suggests that repeated exposure to the Bruce treadmill test may have contributed to the increase in exercise duration. Such improvement may reflect greater familiarity with the testing procedure, improved tolerance of maximal treadmill testing, or other non-specific repeated-measurement effects. Thus, the placebo response provides an empirical indication that part of the observed improvement may have occurred independently of active supplementation.

Nevertheless, a significant treatment condition × measurement time interaction was observed for exercise duration (*p* = 0.002), indicating that the pattern of change differed between the SLAB51 and placebo conditions. Accordingly, the treatment-related finding should be interpreted in terms of this differential response rather than the absolute increase from baseline under SLAB51 alone. Exercise duration continued to increase after 1 week of SLAB51 supplementation, whereas the corresponding improvement under placebo was more limited. This pattern suggests that repeated-test familiarization alone may not fully explain the observed difference between conditions. The additional post hoc crossover analysis estimated a one-week SLAB51–placebo difference of 33.32 s (95% CI: 17.42 to 49.22) after accounting for period, randomized sequence, and within-participant dependence. This supports a treatment-associated difference at one week, although possible carryover and individual familiarization effects cannot be completely excluded.

A similar distinction is important for exercise duration above RER 1.0. Unlike total exercise duration, no significant changes across measurement time points were observed under the placebo condition, whereas a significant treatment condition × measurement time interaction was observed (*p* = 0.004). Within the SLAB51 condition, exercise duration above RER 1.0 increased after 1 week compared with both baseline and single intake. Because a comparable change was not observed under placebo, this pattern provides comparatively stronger evidence of a treatment-associated differential response. The longer exercise duration above RER 1.0 indicates that participants maintained exercise for a longer period after RER exceeded 1.0 during the later portion of the graded treadmill test. This may reflect a greater ability to sustain exercise as physiological and metabolic demands progressively increased. However, because ventilatory threshold was not independently determined, exercise duration above RER 1.0 should not be interpreted as a direct measure of ventilatory threshold.

The interpretation of these performance findings should also consider the absence of a significant treatment-related change in VO_2_max. VO_2_max reflects integrated cardiovascular and skeletal-muscle adaptations that are strongly influenced by long-term training [20,21]. Given the high baseline fitness of the participants and the short supplementation period, a substantial change in maximal aerobic capacity would be difficult to achieve. Thus, the exercise-duration findings raise the possibility that SLAB51 may influence aspects of exercise tolerance or performance sustainability that are not captured by VO_2_max. Exercise tolerance depends not only on maximal oxygen uptake but also on the ability to continue exercise despite progressively increasing physiological and metabolic stress [3,22]. Previous studies have suggested that probiotics, including SLAB51, may influence physiological processes related to metabolic regulation [13,15,16], providing a possible physiological context for the present findings. However, the present study did not directly assess submaximal oxygen utilization, gross exercise efficiency, or muscle oxygenation; therefore, the mechanisms underlying the observed exercise-duration response remain uncertain.

Second, pre-exercise SOD activity showed a significant treatment condition × measurement time interaction (*p* = 0.029). SOD activity increased after 1 week of SLAB51 supplementation, whereas no significant changes across measurement time points were observed under placebo. As with the exercise-performance outcomes, the increase within the SLAB51 condition should not be considered in isolation. Rather, the absence of a comparable placebo response together with the significant interaction indicates a differential response between treatment conditions. This finding suggests that short-term SLAB51 supplementation may be associated with a favorable change in pre-exercise antioxidant-related physiological status.

SOD is an important component of the endogenous antioxidant defense system and contributes to the maintenance of redox homeostasis. This response may be particularly relevant in elite athletes, who are likely to possess relatively well-developed endogenous antioxidant defenses as a result of chronic training. Probiotics have been reported to influence antioxidant and oxidative stress-related pathways through several biological processes [12], providing a plausible physiological context for the observed response. However, the increase in SOD should not be interpreted as direct evidence that enhanced SOD activity caused the improvement in exercise duration or directly delayed fatigue. Rather, the performance response and increased pre-exercise SOD activity should be considered parallel physiological responses observed under the SLAB51 condition. Because SOD was measured before the treadmill test, the present finding reflects pre-exercise antioxidant-related status rather than a direct reduction in exercise-induced oxidative stress.

Third, no significant treatment-related interaction effects were observed for VO_2_max, lactate removal, or MDA concentration. Lactate production and removal are influenced by exercise intensity, accumulated workload, mitochondrial oxidative capacity, and lactate transport [23]. In the present study, participants exercised for a longer duration under the SLAB51 condition, which may have altered the total metabolic load before recovery and complicated the interpretation of post-exercise lactate responses. Accordingly, the present data do not provide evidence that short-term SLAB51 supplementation specifically enhanced post-exercise lactate removal.

MDA reflects lipid peroxidation associated with oxidative stress [24]. Although pre-exercise SOD activity showed a differential increase under the SLAB51 condition, no significant treatment-related interaction was observed for pre-exercise MDA concentration. SOD activity and MDA concentration represent different aspects of redox regulation and may differ in both the magnitude and timing of their physiological responses. Accordingly, the present findings suggest that the short-term antioxidant-related response was more apparent in antioxidant enzyme activity than in circulating lipid-peroxidation markers. Because both SOD and MDA were measured before the treadmill test, these results primarily represent pre-exercise antioxidant and oxidative stress-related status rather than exercise-induced lipid peroxidation or oxidative injury.

An important feature of the present study is that the observed differential responses occurred in elite athletes with high levels of exercise capacity and substantial training-induced physiological adaptation. In highly trained populations, further improvements in physical performance may be relatively difficult to achieve because many physiological systems have already undergone extensive adaptation. Therefore, the differential response in exercise duration, particularly during the later portion of the graded exercise test, may have practical relevance for athletes who are required to sustain performance under progressively increasing physiological stress. The randomized, double-blind, placebo-controlled crossover design also allowed SLAB51 and placebo responses to be evaluated within the same participants, thereby reducing between-subject variability. At the same time, the improvement observed under placebo emphasizes the importance of separating treatment-related responses from familiarization and other non-specific effects of repeated testing.

Several limitations should nevertheless be considered. First, the one-week supplementation period primarily reflects short-term physiological responses and does not allow conclusions regarding longer-term adaptations or persistent changes in the gut microbiota. Gut microbiota composition and microbial metabolites were not directly assessed, and a systematic review of trials in patients with major depressive disorders reported no consistent effects of probiotic supplementation on gut microbiota diversity or composition and insufficient longer-term data [25]. Second, the final analytical sample consisted of 36 participants (Sequence A, *n* = 16; Sequence B, *n* = 20), which was below the initially estimated minimum sample size of 40 because of a higher-than-anticipated rate of attrition and exclusion. This may have reduced statistical power for some outcomes.

Third, although participants were instructed to abstain from vigorous exercise for at least 12 h before each assessment and to arrive at the laboratory in a fasted state, training load in the days preceding each assessment, carbohydrate intake, hydration status, caffeine intake, sleep, and recent competition were not systematically standardized or monitored. These uncontrolled factors may have contributed to variability in physiological and performance responses across repeated assessments. In addition, only one untreated baseline assessment was obtained before Period 1, and no separate pre-intervention baseline assessment was conducted before Period 2. Although the additional post hoc LMM accounted for period and randomized sequence, the absence of a second-period baseline limited the assessment of changes in untreated physiological status before the second intervention.

Furthermore, although a two-week washout period was implemented to minimize residual effects between intervention periods, potential carryover effects cannot be completely excluded. The additional post hoc LMM accounted for period, randomized sequence, and within-participant dependence, providing a more appropriate analysis of the crossover design. Nevertheless, repeated maximal treadmill testing may have produced familiarization effects, as suggested by the improvement observed under placebo. In addition, SOD showed significant period-related variation and departures from residual normality. Therefore, these findings should be interpreted with consideration of the remaining methodological limitations.

Overall, short-term SLAB51 supplementation was associated with differential responses in exercise duration, exercise duration above RER 1.0, and pre-exercise SOD activity compared with placebo. For total exercise duration, improvement was also observed under placebo, indicating that familiarization or other non-specific repeated-testing effects likely contributed to the overall change. Therefore, the treatment-related interpretation is based on the differential pattern between SLAB51 and placebo rather than on the within-SLAB51 improvement alone. Importantly, the additional post hoc LMM analysis also identified a significant treatment-associated difference in total exercise duration after one week (mean difference = 33.32 s, 95% CI: 17.42 to 49.22, *p* < 0.001), even after accounting for period, randomized sequence, and within-participant dependence. This result was consistent with the original ANOVA findings, providing further evidence supporting the potential benefits of SLAB51 supplementation for exercise performance. Furthermore, exercise duration above RER 1.0 and pre-exercise SOD activity showed significant treatment × time interactions without comparable significant changes under placebo, and the additional LMM analysis also identified significant treatment differences after one week, providing further exploratory evidence of favorable treatment-associated responses. Further adequately powered crossover studies incorporating period-specific baseline assessments, longer intervention periods, and additional physiological and microbiome-related assessments are needed to confirm these findings and clarify the mechanisms underlying these responses.

## 5. Conclusions

One week of SLAB51 supplementation was associated with longer total exercise duration and exercise duration above RER 1.0, as well as higher pre-exercise SOD activity compared with placebo. Importantly, the additional post hoc LMM analysis identified a significant treatment-associated difference in total exercise duration, the primary outcome, after one week (mean difference = 33.32 s, 95% CI: 17.42 to 49.22, *p* < 0.001), even after accounting for period, randomized sequence, and within-participant dependence. Significant differences in the secondary outcomes were also observed after one week, whereas no significant treatment differences were observed after a single intake. However, the improvement observed under placebo, the absence of significant treatment condition × measurement time interactions for VO_2_max and MDA in the original ANOVA, and the potential influence of familiarization, period, and carryover effects warrant cautious interpretation. Further adequately powered crossover studies incorporating period-specific baseline assessments are needed to confirm these findings and clarify the underlying mechanisms.

## Figures and Tables

**Table 1:** Functional endpoints.

Category	Evaluation Items	Measurement Tools
Exercise performance indicators	VO_2_max, exercise duration, exercise duration above RER 1.0	Treadmill (Bruce protocol)
Antioxidant and oxidative stress indicators	Superoxide dismutase (SOD) and Malondialdehyde (MDA)	Venous blood collection
Blood lactate accumulation	Blood lactate concentration	YSI 1500 Sport

**Table 2:** Physical and physiological characteristics of the participants in the final analysis sample, presented as mean ± standard deviation for the total sample and separately by treatment sequence. No between-sequence hypothesis tests were performed.

Variable	Total (*n* = 36)	Sequence A (*n* = 16)	Sequence B (*n* = 20)
Age (years)	20.95 ± 1.25	21.19 ± 1.42	20.75 ± 1.09
Height (cm)	180.21 ± 7.79	179.58 ± 7.35	180.72 ± 8.27
Body mass (kg)	75.14 ± 10.64	73.43 ± 11.10	76.50 ± 10.34
BMI (kg/m^2^)	23.09 ± 2.52	22.69 ± 2.28	23.41 ± 2.71
Systolic blood pressure (mmHg)	128.86 ± 14.20	126.13 ± 15.68	131.05 ± 12.88
Diastolic blood pressure (mmHg)	73.60 ± 6.94	74.50 ± 7.82	72.88 ± 6.26
Resting heart rate (beats/min)	67.06 ± 9.06	64.75 ± 7.24	68.90 ± 10.08
Body temperature (°C)	36.55 ± 0.26	36.52 ± 0.16	36.58 ± 0.32

**Table 3:** Results of a two-way repeated-measures ANOVA for exercise duration.

Variable	Group	Baseline	Single Intake	1-Week Intake	Factor	*F*-Value	*p*-Value (ηp^2^)
Exercise duration (s)	SLAB51	886.28 ± 72.04	918.06 ± 60.33	953.94 ± 82.89	Group	17.078	<0.001 (0.328)
	Placebo		914.36 ± 66.91	921.33 ± 72.18	Time	26.541	<0.001 (0.431)
					Group × Time	8.760	0.002 (0.200)

Results are expressed as mean ± SD. The baseline value represents a single untreated assessment obtained before Period 1 and served as the common baseline for both treatment conditions. No separate pre-intervention baseline was obtained before Period 2.

**Table 4:** Results of post hoc tests for the group × time interaction effect on exercise duration. The numbers in parentheses refer to the *p*-values calculated in the specific comparisons between time points.

Variable	Group	Baseline vs. Single Intake	Baseline vs. 1-Week Intake	Single Intake vs. 1-Week Intake
Exercise duration (s)	SLAB51	31.77 (0.001)	67.66 (0.001)	35.88 (0.004)
	Placebo	28.08 (0.001)	35.05 (0.001)	6.97 (0.088)

**Table 5:** Results of a two-way repeated-measures ANOVA for exercise duration above RER 1.0.

Variable	Group	Baseline	Single Intake	1-Week Intake	Factor	*F*-Value	*p*-Value (ηp^2^)
Exercise duration above RER 1.0 (s)	SLAB51	232.06 ± 105.46	258.42 ± 109.93	316.25 ± 114.90	Group	3.455	0.071 (0.090)
	Placebo		254.53 ± 98.92	258.78 ± 89.10	Time	5.929	0.006 (0.145)
					Group × Time	6.005	0.004 (0.146)

Results are expressed as mean ± SD. The baseline value represents a single untreated assessment obtained before Period 1 and served as the common baseline for both treatment conditions. No separate pre-intervention baseline was obtained before Period 2.

**Table 6:** Results of post hoc tests for the group × time interaction effect on exercise duration above RER 1.0. The numbers in parentheses refer to the *p*-values calculated in the specific comparisons between time points.

Variable	Group	Baseline vs. Single Intake	Baseline vs. 1-Week Intake	Single Intake vs. 1-Week Intake
Exercise duration above RER 1.0 (s)	SLAB51	26.36 (0.192)	84.19 (0.001)	57.83 (0.004)
	Placebo	22.47 (0.206)	26.72 (0.191)	4.25 (0.759)

**Table 7:** Results of a two-way repeated-measures ANOVA for VO_2_max.

Variable	Group	Baseline	Single Intake	1-Week Intake	Factor	*F*-Value	*p*-Value (ηp^2^)
VO_2_max (mL/min/kg)	SLAB51	55.08 ± 5.49	56.50 ± 6.03	53.80 ± 6.34	Group	0.008	0.928 (<0.001)
	Placebo		55.26 ± 5.17	54.92 ± 6.62	Time	2.041	0.141 (0.055)
					Group × Time	2.018	0.159 (0.055)

Results are expressed as mean ± SD. The baseline value represents a single untreated assessment obtained before Period 1 and served as the common baseline for both treatment conditions. No separate pre-intervention baseline was obtained before Period 2.

**Table 8:** Results of a two-way repeated-measures ANOVA for SOD.

Variable	Group	Baseline	Single Intake	1-Week Intake	Factor	*F*-Value	*p*-Value (ηp^2^)
SOD (U/mL)	SLAB51	1.43 ± 0.21	1.34 ± 0.31	1.77 ± 0.67	Group	1.338	0.255 (0.037)
	Placebo		1.39 ± 0.31	1.50 ± 0.34	Time	14.235	<0.001 (0.289)
					Group × Time	3.722	0.029 (0.096)

Results are expressed as mean ± SD. The baseline value represents a single untreated assessment obtained before Period 1 and served as the common baseline for both treatment conditions. No separate pre-intervention baseline was obtained before Period 2.

**Table 9:** Results of post hoc tests for the group × time interaction effect on SOD. The numbers in parentheses refer to the *p*-values calculated in the specific comparisons between time points.

Variable	Group	Baseline vs. Single Intake	Baseline vs. 1-Week Intake	Single Intake vs. 1-Week Intake
SOD (U/mL)	SLAB51	−0.08 (0.167)	0.34 (0.003)	0.42 (0.001)
	Placebo	−0.03 (0.571)	0.07 (0.336)	0.11 (0.059)

**Table 10:** Results of a two-way repeated-measures ANOVA for MDA.

Variable	Group	Baseline	Single Intake	1-Week Intake	Factor	*F*-Value	*p*-Value (ηp^2^)
MDA (pmol/mL)	SLAB51	36.54 ± 6.31	53.23 ± 25.37	49.95 ± 16.71	Group	0.005	0.947 (<0.001)
	Placebo		47.82 ± 21.17	55.76 ± 21.17	Time	35.205	<0.001 (0.501)
					Group × Time	1.204	0.290 (0.033)

Results are expressed as mean ± SD. The baseline value represents a single untreated assessment obtained before Period 1 and served as the common baseline for both treatment conditions. No separate pre-intervention baseline was obtained before Period 2.

**Table 11:** Results of a two-way repeated-measures ANOVA for lactate removal (lactate reduction).

Variable	Group	Baseline	Single Intake	1-Week Intake	Factor	*F*-Value	*p*-Value (ηp^2^)
Lactate removal (%)	SLAB51	86.37 ± 18.55	71.89 ± 10.56	68.67 ± 11.81	Group	4.060	0.052 (0.104)
	Placebo		74.78 ± 12.45	72.70 ± 10.78	Time	18.197	<0.001 (0.342)
					Group × Time	1.137	0.318 (0.031)

Results are expressed as mean ± SD. The baseline value represents a single untreated assessment obtained before Period 1 and served as the common baseline for both treatment conditions. No separate pre-intervention baseline was obtained before Period 2.

**Table 12:** Post hoc linear mixed-effects model treatment contrasts for the principal outcomes.

Outcome	Assessment Time	Mean Difference	95% CI	*p*-Value	Interaction *p*
Exercise duration (s)	Single intake	3.14	−12.77 to 19.04	0.696	0.009
	1-Week intake	33.32	17.42 to 49.22	<0.001	
Duration above RER 1.0 (s)	Single intake	1.69	−36.58 to 39.97	0.930	0.045
	1-Week intake	56.99	18.71 to 95.26	0.004	
SOD (U/mL)	Single intake	−0.075	−0.237 to 0.087	0.361	0.020
	1-Week intake	0.199	0.037 to 0.361	0.017	

Values represent adjusted treatment differences (SLAB51 − placebo). CI, confidence interval; Interaction *p*, treatment × time interaction. No joint multiplicity adjustment was applied across time points or outcomes.

## Data Availability

The datasets generated and/or analyzed during the current study are not publicly available due to participant privacy and institutional restrictions but are available from the corresponding author on reasonable request.

## Abbreviations

The following abbreviations are used in this manuscript:

ATP	Adenosine Triphosphate
BMI	Body Mass Index
CFU	Colony-Forming Units
FiO_2_	Fraction of Inspired Oxygen
HIF-1α	Hypoxia-Inducible Factor-1 Alpha
MDA	Malondialdehyde
Nrf2	Nuclear Factor Erythroid-2-Related Factor 2
RER	Respiratory Exchange Ratio
ROS	Reactive Oxygen Species
SaO_2_	Arterial Oxygen Saturation
SOD	Superoxide Dismutase
VO_2_max	Maximal Oxygen Uptake
